# The effect of matrices on the gene expression profile of patient-derived head and neck carcinoma cells for in vitro therapy testing

**DOI:** 10.1186/s12935-023-02982-y

**Published:** 2023-07-24

**Authors:** Aini Hyytiäinen, Katja Korelin, Mervi Toriseva, Tommy Wilkman, Satu Kainulainen, Karri Mesimäki, Johannes Routila, Sami Ventelä, Heikki Irjala, Matthias Nees, Ahmed Al-Samadi, Tuula Salo

**Affiliations:** 1 Department of Oral and Maxillofacial Diseases, University of Helsinki, Helsinki, Finland; 2 Translational Immunology Research Program, Faculty of Medicine, University of Helsinki, Helsinki, Finland; 3 Institute of Biomedicine, University of Turku, Turku, 20520 Finland; 4 FICAN West Cancer Centre, University of Turku and Turku University Hospital, Turku, Finland; 5 Department of Oral and Maxillofacial Diseases, Helsinki University Hospital, Helsinki, Finland; 6 Department of Otorhinolaryngology – Head and Neck surgery, Turku University Hospital and University of Turku, Turku, Finland; 7 Turku Bioscience Centre, University of Turku and Åbo Akademi University, Turku, Finland; 8 Institute of Dentistry, School of Medicine, University of Eastern Finland, Kuopio Campus, Kuopio, Finland; 9 Research Unit of Population Health, Faculty of Medicine, University of Oulu, Oulu, Finland; 10 Medical Research Center, Oulu University Hospital, Oulu, Finland; 11 Department of Pathology, Helsinki University Hospital (HUS), Helsinki, Finland

**Keywords:** Head and neck cancer, Patient-derived cells, Gene expression, In vitro 3D, Tumor microenvironment

## Abstract

**Objective:**

Head and neck squamous cell carcinoma (HNSCC) is a highly aggressive tumor with a 5-year mortality rate of ~ 50%. New in vitro methods are needed for testing patients’ cancer cell response to anti-cancer treatments. We aimed to investigate how the gene expression of fresh carcinoma tissue samples and freshly digested single cancer cells change after short-term cell culturing on plastic, Matrigel or Myogel. Additionally, we studied the effect of these changes on the cancer cells’ response to anti-cancer treatments.

**Materials/methods:**

Fresh tissue samples from HNSCC patients were obtained perioperatively and single cells were enzymatically isolated and cultured on either plastic, Matrigel or Myogel. We treated the cultured cells with cisplatin, cetuximab, and irradiation; and performed cell viability measurement. RNA was isolated from fresh tissue samples, freshly isolated single cells and cultured cells, and RNA sequencing transcriptome profiling and gene set enrichment analysis were performed.

**Results:**

Cancer cells obtained from fresh tissue samples changed their gene expression regardless of the culturing conditions, which may be due to the enzymatic digestion of the tissue. Myogel was more effective than Matrigel at supporting the upregulation of pathways related to cancer cell proliferation and invasion. The impacts of anti-cancer treatments varied between culturing conditions.

**Conclusions:**

Our study showed the challenge of in vitro cancer drug testing using enzymatic cell digestion. The upregulation of many targeted pathways in the cultured cells may partially explain the common clinical failure of the targeted cancer drugs that pass the in vitro testing.

## Introduction

Head and neck cancers are a heterogeneous group of highly aggressive tumors, of which 90% are squamous cell carcinomas (HNSCCs) [1]. HNSCC is the eighth most common cancer worldwide [2]. Despite improvement in the clinical outcome of many tumor types, the overall 5-year survival rate of HNSCC remains around 50%, mainly due to poor availability of effective therapeutic options for these patients [3]. The primary treatment for HNSCC is surgery combined with radio- and/or chemoradiotherapy [4]. Other FDA-approved treatment options include the targeted therapy drug cetuximab and immune checkpoint inhibitors pembrolizumab and nivolumab [4]. However, current treatment protocols are associated with significant toxicity, and many patients develop treatment resistance and cancer recurrence [5].

The tumor microenvironment (TME) consists of all the non-malignant cellular and acellular components surrounding the tumor, including stromal cells, such as immune cells, cancer-associated fibroblasts (CAFs), blood vessels, extracellular matrix (ECM), and signaling molecules [6]. HNSCC cells are notably affected by their TME, and it plays a major role in disease progression and patient prognosis [7]. The ECM is a significant component of the TME, and it includes e.g. different kinds of proteins, proteoglycans, and polysaccharides [8]. The ECM in solid tumors differs significantly from that in normal organs and could have a direct effect on the cancer cells response to anticancer therapy [9].

Traditionally, in vitro studies with cancer cells are done in 2D plastic wells. But the artificial nature of the culturing conditions has been shown to poorly represent the 3D nature of the solid tumor in the body, and the vital interaction with the human TME is missing [10]. Thus, in recent years, 3D cell cultures with different ECMs have been developed to better mimic the in vivo condition and to give more reliable results for the in vitro studies. Most of these matrices are extracted from animals (like mouse tumor derived Matrigel) or derived from synthetic materials. Our group has invented the first human tumor-derived matrix, Myogel, which is extracted from uterus leiomyomas, and its proteome differs significantly from Matrigel [11]. We have shown that Myogel enhances the proliferation of freshly isolated cancer cells from primary tumors compared to plastic and Matrigel [12]. Myogel also improved the reliability of HNSCC drug testing [13]. Our recent publication showed that the selection of matrix type for cell culture experiments affects several genes and pathways, and plays a significant role in HNSCC cell lines phenotype [14].

Here, we aimed to investigate how the RNA transcriptome profiles of fresh carcinoma tissue samples and isolated cancer cells change after culturing the cells using RNA sequencing transcriptome profiling. Additionally, we studied the effect of these changes on the cancer cells response to anti-cancer treatments.

## Materials and methods

### Patients’ samples

The HNSCC patient samples were collected from the Helsinki University Central Hospital according to the Ethical Committee of the Northern Ostrobothnia Hospital District, Finland (statement number 31/2016) approval, and from Turku University Hospital approved by the regional ethics committee of the Hospital District of Southwest Finland Turku (Dnro 166/1801/2015). The study was carried out according to the Declaration of Helsinki. Patient participation in the study was voluntary and they all signed consent forms. The clinical and pathological characteristics of the patients are presented in Table 1. The fresh tissue samples were obtained intraoperative and placed in ice-cold Hank’s Balanced Salt Solution (HBSS; supplied with 100 U/ml penicillin, 100 µg/ml streptomycin, and 250ng/ml amphotericin B (Thermo Scientific, Massachusetts, USA). The samples were taken from the area adjacent to the center of the tumor to assure the presence of the carcinoma tissue cells, including mostly carcinoma cells and some cancer-associated fibroblasts. Each tissue sample was placed in a Petri dish containing HBSS and kept on ice.

**Table 1:** Clinical and pathological characteristics of the obtained patients’ samples

Patient number	Patient code	Sex^a^	Age^b^	TNM (8th edition) (clinical/pathological)	Specimen site	Type^c^	Grade
1	UH-SCC-17 A	M	50	cT3N2b	Mobile tongue	Pri	G2
2	UT-HNC-23-T	M	66	cT4aN0M0/pT4a	Gingiva	Pri	G3
3	UH-SCC-18 A	M	51	cT3N3bM0	Mobile tongue	Pri	G2
4	UH-SCC-6	F	73	cT3N0M0/pT3pN3b M0	Lower gum	Pri	G3
5	UH-SCC-8	F	80	cT2N0M0/pT3pN0M	Buccal mucosa	Pri	G2
6	UH-SCC-12	F	44	cT2N0M0	Mobile tongue	Pri	G2
7	UH-SCC-14	F	68	cT4aN0M0	Unspecific parts of mouth	Pri	G1

^a^M = male, F = female, ^b^Age in years, ^c^Pri = primary tumor

Each sample was cut into two pieces. The first piece was snap frozen in liquid nitrogen, stored at − 80 °C and later lysed in RLT-buffer (miRNA RNeasy Kits, Qiagen, Düsseldorf, Germany) for RNA isolation. The second piece was used for isolating single cancer cells according to the following protocol: necrotic tissue was removed using a scalpel, and vital tissue pieces were placed into a new Petri dish containing HBSS and minced into small (1–2 mm) pieces with a scalpel. The tissue pieces were transferred to a 15 ml falcon tube and centrifuged for 5 min at 1000 rpm at 4 °C. The supernatant was discarded, and a fresh HBSS buffer was added before another round of centrifugation. The tissue pellet was suspended in a 5ml HBSS buffer containing 1 mg/ml collagenase type I from Clostridium histolyticum (Sigma-Aldrich, St. Louis, Mo, USA) and placed on a rocker platform at 37 °C. After 2 h, the tube was centrifuged, and the supernatant was discarded and replaced with a fresh HBSS buffer and centrifuged for another round. The digested sample was suspended in a HBSS buffer, filtered using a 100 μm cell strainer (Falcon Cell Stainer, Fisher Scientific, Portsmouth, NH, USA) and the flow-through was collected and centrifuged. The supernatant was discarded, and the cell pellet was suspended in MEM media (MEM; Gibco, Waltham, MA, USA) supplemented with 10% heat-inactivated FBS (Gibco), a 1% nonessential amino acid solution (Gibco), 2 mM glutamine, 100 U/ml penicillin, 100 µg/ml streptomycin and 250 ng/ml amphotericin B (all from Sigma-Aldrich). Isolated single cells were divided into two groups. The first group was put directly in RLT-buffer and stored at − 80 °C for later use and the second group was cultured on different matrices.

### Culturing conditions and anticancer compounds

Isolated single cells were cultured on 96-well plates (PerkinElmer, Waltham, MA, USA) using three different culturing conditions: on plastic, on Matrigel (Corning, Corning, NY, USA), or on Myogel coated wells [11].

Myogel and Matrigel were thawed overnight on ice (4 °C). We pre-chilled pipette tips and other equipment in a freezer (–20 °C) and 96-well plates on ice. Matrigel and Myogel were diluted with a cell culture media to a final concentration of 0.5 mg/ml. Matrices (50 µL) were added to 96-well plates, and plates were left overnight in a cell culture incubator. Only the cell culture media was added to the uncoated wells. On the following day, the freshly isolated cancer cells were counted using the Scepter™ 2.0 Cell Counter (Merck Millipore, Burlington, MA, USA) and suspended to 1000–3000 cells/well. Plates were returned to the incubator overnight (37 °C, 5% CO_2_, 95% humidity), and drugs were added on the following day. We treated the cells with the targeted therapy drug cetuximab (5 µg/ml), chemotherapy drug cisplatin (0.5 µg/ml), and/or irradiation (the irradiation dose as fractions, 2 Gy/day for three days) using gamma irradiator OB29/4 (STS, Braunschweig, Germany).

### Cell viability

We used CellTiter-Glo (CTG) Luminescent Cell Viability Assay (Promega, Madison, WI, USA) for cell viability analysis. After three days of incubation, the plates were taken to room temperature for 15 min before 100 µL of CTG was dispensed in each well. The plates were put on a plate shaker (Heidolph, Schwabach, Germany) for 5 min at 450 rpm and after that the plates were spun for 5 min at 1000 rpm. Finally, the plates were placed in the BMG PHERAstar FS (BMG Labtech, Offenburg, Germany) plate reader to detect cell viability. The Luminescent Cell Viability Assay was repeated after freezing and thawing freshly isolated single cells treated with the same anticancer compounds and/or irradiation, as described above. In this experiment, after single cell isolation, cells were cultured until 80–90% confluence was reached and then frozen using 90% FBS and 10% DMSO. The cells were kept in liquid nitrogen for 3–4 weeks, before thawing the cells and conducting the second treatment testing.

### RNA sequencing and data analysis

Patient samples UH-SCC-17 A, UH-SCC-18 A, and UT-HNC-23-T were used to study the effect of the culturing conditions on the cancer cell gene expression profile using RNA sequencing transcriptome profiles. RNA was isolated from the fresh tissue samples, freshly isolated single cells, and cells cultured on plastic, Matrigel and Myogel for three days, for the UH-SCC-17 A, UH-SCC-18 A samples, and five days, for the UT-HNC-23-T sample. Total RNA was extracted from cultured cells, frozen fresh tissue samples, and single cells using miRNeasy Tissue/Cells Advanced Mini Kit (Qiagen) according to manufacturer instructions. If some clots or fragments of gels existed in the cell lysate, sonication was used to solubilize them. The RNA was purified with Zymo RNA Clean & Concentrator-5 (Nordic BioSite, Sweden) according to manufacturer instructions. The quality of total RNA was assessed with a TapeStation (Agilent Technologies, Santa Clara, CA, USA), and only samples of high quality (RNA integrity value > 8) were included in the analyses. Samples were sequenced with Illumina NextSeq 500 sequencer (Illumina, San Diego, CA, USA) in two High output runs using Illumina Stranded Total RNA Prep with Ribo-Zero Plus -kit. The sequencing was performed as single-end sequencing for read length 75 bp (SE75 or 1 × 75 bp).

The sequencing reads were aligned against the Genome Reference Consortium Human Build 38 patch release 13 (GRCh38.p13, GCA_000001405.28) reference using the Spliced Transcripts Alignment to a Reference (STAR v. 2.7.6a) tool. Alignment statistics were collected with the QualiMap tool (v.2.2.1). Genes were annotated against GENCODE human release 38 and the featureCounts software (from Subread-package v.2.0.1) was used to produce the gene counts for each sample. Differentially expressed genes between experimental groups were called using DESeq2 package in R environment. The principal component analysis (PCA) plots were also drawn to visualize the sample clustering behaviour. Finally, to connect gene expression signatures with previously known gene sets, a gene set enrichment analysis (GSEA, https://www.gsea-msigdb.org/gsea/index.jsp) [15] was performed. Genes were ranked using signal-to-noise ratio and gene set permutation was used for FDR estimation and enrichment score adjustment. All analyses were performed by the Functional Genomics Unit (FuGU) at the University of Helsinki.

### Statistical analysis

Values are provided as mean ± standard deviation. All statistical analyses were performed using SPSS (IBM SPSS Statistics, version 28.0; Armonk NY, IBM Corp.) We performed a one-way analysis of variance (ANOVA) followed by Bonferroni correction to determine statistical significance. We set statistical significance to p < 0.05. P values were presented as follows: * = P ≤ 0.05, ** = P ≤ 0.01, *** P ≤ 0.001. OriginLab (OriginLab, Northampton, Massachusetts, USA) software was used to create the graphs.

## Results

### Culturing patient-derived cancer cells changes their transcriptomic profile in all culturing conditions

**Fig. 1:**
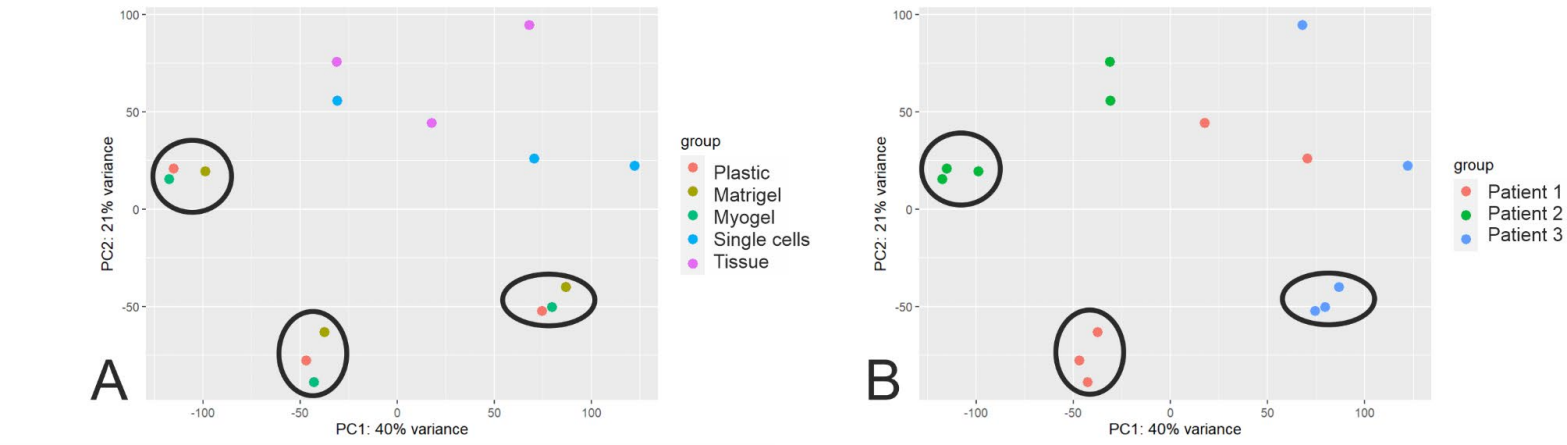
**Patient-derived cells cultured in different culturing conditions clustered far away from fresh tissue sample and digest single cells**. (**A**) Principal component analysis (PCA) shows that cells obtained from fresh tissue samples from HNSCC patients changed their transcriptomic profile regardless of the culturing conditions. (**B**) PCA projection of the three patient samples show that each sample clustered far away from fresh tissue samples and single cells

To demonstrate how culturing conditions affect cancer cell gene expression profiles and what condition would be the best to preserve the original cell gene expression profile, we performed RNA transcriptome analysis. We hypothesized that culturing cells on matrix, especially human tumor-derived matrix Myogel, would have a better result than plastic alone in preserving the transcriptomic profile of cells. Contrary to our hypothesis, the results revealed that transcriptomic profiles changed remarkably after culturing the tumor-derived primary cells, regardless of the culturing condition, when compared with fresh tissue and the digested single cells (Fig. 1). For all three patients, cultured cells on plastic, Myogel, and Matrigel, clustered together in the PCA far from the fresh tissue and the digested single cells (Fig. 1A). This clustering was also clearly driven by the patient-specific samples (Fig. 1B).

**Fig. 2:**
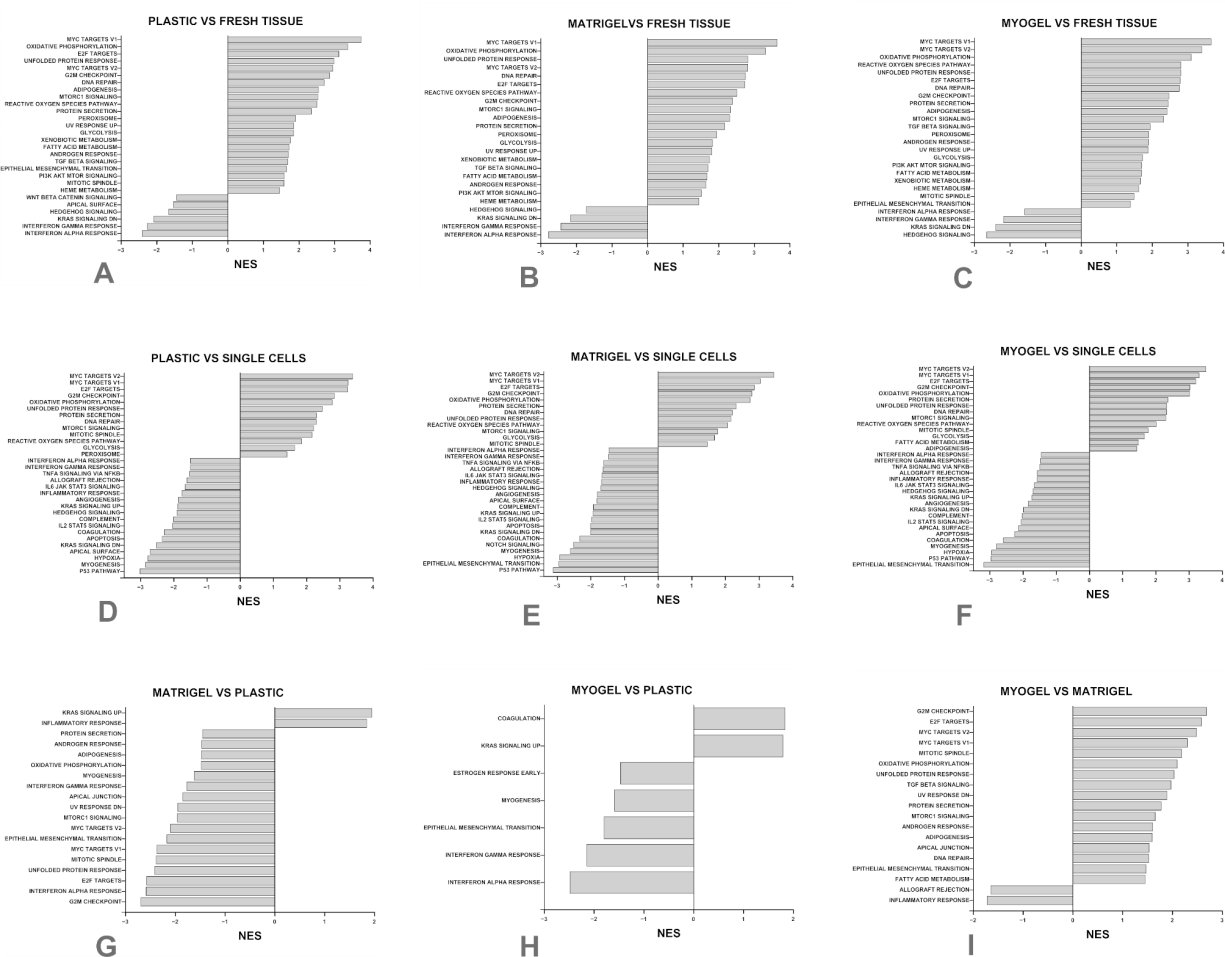
**Patient-derived cells cultured in different culturing conditions express significantly up- and downregulated pathways.** Results of the gene set enrichment analysis (GSEA) show up- and downregulated pathways in patient-derived cells cultured in different matrices. (**A**) Plastic in comparison with fresh tissue sample, (**B**) Matrigel in comparison with fresh tissue sample, (**C**) Myogel in comparison with fresh tissue sample, (**D**) Plastic in comparison with freshly digested single cells, (**E**) Matrigel in comparison with freshly digested single cells, (**F**) Myogel in comparison with freshly digested single cells, (**G**) Matrigel in comparison with plastic, (**H**) Myogel in comparison with plastic, (**I**) Myogel in comparison with Matrigel. NES, normalized enrichment score

Gene ontology enrichment analysis showed that several hallmark pathways were up- and downregulated in cultured cells (on plastic, Matrigel, and Myogel) compared with fresh tissue samples (Table 2; Fig. 2A-C). The majority of the upregulated pathways were shared in all culturing conditions (20/22 in plastic, 20/20 in Matrigel, and 20/20 in Myogel). Of the downregulated pathways, there were only four that were statistically significant in Matrigel and Myogel compared to fresh tissue sample: interferon alpha response, interferon gamma response, KRAS signaling DN and Hedgehog signaling. In plastic there were six significantly downregulated pathways compared to fresh tissue sample (Table 2**).**

**Table 2:** **Significantly expressed hallmark pathways in all culturing conditions compared to fresh tissue sample.** Results of the gene set enrichment analysis (GSEA) shows the significantly expressed hallmark pathways in three culturing conditions (plastic, Matrigel, and Myogel) compared to fresh tissue sample. The pathways that passed the filter criteria had a FDR q-val < 0.05. ES, enrichment score; NES, normalized enrichment score

MATRIGEL VS FRESH TISSUE									
UPREGULATED					DOWNREGULATED				
HALLMARK PATHWAY	ES	NES	NOM p-val	FDR q-val	HALLMARK PATHWAY	ES	NES	NOM p-val	FDR q-val
MYC TARGETS V1	0.69	3.75	0.000	0.00	INTERFERON ALPHA RESPONSE	-0.54	-2.41	0.000	0.00
OXIDATIVE PHOSPHORYLATION	0.62	3.37	0.000	0.00	INTERFERON GAMMA RESPONSE	-0.46	-2.27	0.000	0.00
E2F TARGETS	0.57	3.12	0.000	0.00	KRAS SIGNALING DN	-0.44	-2.09	0.000	0.00
UNFOLDED PROTEIN RESPONSE	0.60	2.98	0.000	0.00	HEDGEHOG SIGNALING	-0.47	-1.66	0.007	0.01
MYC TARGETS V2	0.67	2.95	0.000	0.00	APICAL SURFACE	-0.41	-1.53	0.027	0.03
G2M CHECKPOINT	0.53	2.86	0.000	0.00	WNT BETA CATENIN SIGNALING	-0.39	-1.44	0.047	0.05
DNA REPAIR	0.52	2.70	0.000	0.00					
ADIPOGENESIS	0.47	2.54	0.000	0.00					
MTORC1 SIGNALING	0.47	2.53	0.000	0.00					
REACTIVE OXYGEN SPECIES PATHWAY	0.59	2.51	0.000	0.00					
PROTEIN SECRETION	0.49	2.34	0.000	0.00					
PEROXISOME	0.40	1.91	0.000	0.00					
UV RESPONSE UP	0.35	1.85	0.000	0.00					
GLYCOLYSIS	0.34	1.85	0.000	0.00					
XENOBIOTIC METABOLISM	0.33	1.76	0.000	0.00					
FATTY ACID METABOLISM	0.33	1.72	0.000	0.00					
ANDROGEN RESPONSE	0.36	1.70	0.003	0.00					
TGF BETA SIGNALING	0.39	1.68	0.000	0.00					
EPITHELIAL MESENCHYMAL TRANSITION	0.30	1.65	0.000	0.00					
PI3K AKT MTOR SIGNALING	0.32	1.58	0.003	0.01					
MITOTIC SPINDLE	0.29	1.58	0.000	0.01					
HEME METABOLISM	0.27	1.45	0.003	0.02					
**MATRIGEL VS FRESH TISSUE**									
**UPREGULATED**					**DOWNREGULATED**				
**HALLMARK PATHWAY**	**ES**	**NES**	**NOM p-val**	**FDR q-val**	**HALLMARK PATHWAY**	**ES**	**NES**	**NOM p-val**	**FDR q-val**
MYC TARGETS V1	0.68	3.64	0.000	0.00	INTERFERON ALPHA RESPONSE	-0.64	-2.78	0.0000	0.00
OXIDATIVE PHOSPHORYLATION	0.62	3.31	0.000	0.00	INTERFERON GAMMA RESPONSE	-0.51	-2.44	0.0000	0.00
UNFOLDED PROTEIN RESPONSE	0.57	2.82	0.000	0.00	KRAS SIGNALING DN	-0.46	-2.17	0.0000	0.00
MYC TARGETS V2	0.65	2.82	0.000	0.00	HEDGEHOG SIGNALING	-0.49	-1.72	0.0034	0.00
DNA REPAIR	0.53	2.75	0.000	0.00					
E2F TARGETS	0.51	2.73	0.000	0.00					
REACTIVE OXYGEN SPECIES PATHWAY	0.60	2.51	0.000	0.00					
G2M CHECKPOINT	0.44	2.38	0.000	0.00					
MTORC1 SIGNALING	0.44	2.33	0.000	0.00					
ADIPOGENESIS	0.43	2.31	0.000	0.00					
PROTEIN SECRETION	0.46	2.16	0.000	0.00					
PEROXISOME	0.40	1.94	0.000	0.00					
GLYCOLYSIS	0.34	1.82	0.000	0.00					
UV RESPONSE UP	0.35	1.80	0.000	0.00					
XENOBIOTIC METABOLISM	0.33	1.74	0.000	0.00					
TGF BETA SIGNALING	0.40	1.70	0.003	0.00					
FATTY ACID METABOLISM	0.32	1.67	0.000	0.00					
ANDROGEN RESPONSE	0.34	1.64	0.000	0.00					
PI3K AKT MTOR SIGNALING	0.31	1.51	0.010	0.01					
HEME METABOLISM	0.27	1.44	0.004	0.02					
**MYOGEL VS FRESH TISSUE**									
**UPREGULATED**					**DOWNREGULATED**				
**HALLMARK PATHWAY**	**ES**	**NES**	**NOM p-val**	**FDR q-val**	**HALLMARK PATHWAY**	**ES**	**NES**	**NOM p-val**	**FDR q-val**
MYC TARGETS V1	0.68	3.65	0.000	0.00	INTERFERON ALPHA RESPONSE	-0.59	0.00	0.000	0.00
OXIDATIVE PHOSPHORYLATION	0.63	3.39	0.000	0.00	INTERFERON GAMMA RESPONSE	-0.48	0.00	0.000	0.00
E2F TARGETS	0.56	3.09	0.000	0.00	KRAS SIGNALING DN	-0.45	0.00	0.000	0.00
MYC TARGETS V2	0.65	2.80	0.000	0.00	HEDGEHOG SIGNALING	-0.44	0.02	0.022	0.02
UNFOLDED PROTEIN RESPONSE	0.57	2.80	0.000	0.00					
G2M CHECKPOINT	0.52	2.78	0.000	0.00					
DNA REPAIR	0.53	2.76	0.000	0.00					
ADIPOGENESIS	0.46	2.47	0.000	0.00					
MTORC1 SIGNALING	0.45	2.45	0.000	0.00					
REACTIVE OXYGEN SPECIES PATHWAY	0.59	2.41	0.000	0.00					
PROTEIN SECRETION	0.49	2.32	0.000	0.00					
PEROXISOME	0.40	1.94	0.000	0.00					
UV RESPONSE UP	0.36	1.89	0.000	0.00					
ANDROGEN RESPONSE	0.39	1.89	0.000	0.00					
GLYCOLYSIS	0.35	1.88	0.000	0.00					
TGF BETA SIGNALING	0.40	1.72	0.000	0.00					
PI3K AKT MTOR SIGNALING	0.35	1.70	0.000	0.00					
FATTY ACID METABOLISM	0.33	1.70	0.000	0.00					
XENOBIOTIC METABOLISM	0.31	1.67	0.000	0.00					
HEME METABOLISM	0.31	1.62	0.000	0.00					

When cultured cells were compared with the freshly digested single cells several pathways were affected, and interestingly most of these pathways were shared between all three culturing conditions (Table 3; Fig. 2D-F). Most of the pathways found here were also found in the comparison between cultured cells and fresh tissue. When compared, freshly isolated single cells with fresh tissue several pathways were upregulated, most likely as a result of tissue structure disruption and intracellular dissociation due to enzymatic digestion (Table 4**).**

**Table 3:** **Significantly expressed hallmark pathways in all three culturing conditions compared to single cells.** Results of the gene set enrichment analysis (GSEA) showing the significantly expressed hallmark pathways in three culturing conditions (plastic, Matrigel, and Myogel) compared to single cells. At the end of the table the significantly expressed pathways not in common with all conditions are listed. The pathways that passed the filter criteria had a FDR q-val < 0.05. ES, enrichment score; NES, normalized enrichment score

MATRIGEL VS SINGLE CELLS									
UPREGULATED					DOWNREGULATED				
HALLMARK PATHWAY	ES	NES	NOM p-val	FDR q-val	HALLMARK PATHWAY	ES	NES	NOM p-val	FDR q-val
MYC TARGETS V1	0.65	3.38	0.000	0.00	INTERFERON GAMMA RESPONSE	-0.71	-3.02	0.000	0.00
E2F TARGETS	0.62	3.25	0.000	0.00	TNFA SIGNALING VIA NFKB	-0.67	-2.85	0.000	0.00
G2M CHECKPOINT	0.60	3.23	0.000	0.00	INTERFERON ALPHA RESPONSE	-0.72	-2.79	0.000	0.00
MYC TARGETS V2	0.66	2.83	0.000	0.00	ALLOGRAFT REJECTION	-0.65	-2.71	0.000	0.00
OXIDATIVE PHOSPHORYLATION	0.53	2.77	0.000	0.00	INFLAMMATORY RESPONSE	-0.59	-2.53	0.000	0.00
UNFOLDED PROTEIN RESPONSE	0.50	2.47	0.000	0.00	IL6 JAK STAT3 SIGNALING	-0.63	-2.35	0.000	0.00
MTORC1 SIGNALING	0.43	2.29	0.000	0.00	KRAS SIGNALING UP	-0.54	-2.29	0.000	0.00
PROTEIN SECRETION	0.49	2.29	0.000	0.00	COMPLEMENT	-0.48	-2.04	0.000	0.00
DNA REPAIR	0.43	2.21	0.000	0.00	IL2 STAT5 SIGNALING	-0.47	-2.01	0.000	0.00
MITOTIC SPINDLE	0.41	2.16	0.000	0.00	APOPTOSIS	-0.46	-1.91	0.000	0.00
GLYCOLYSIS	0.34	1.85	0.000	0.00	COAGULATION	-0.47	-1.88	0.000	0.00
REACTIVE OXYGEN SPECIES PATHWAY	0.40	1.64	0.004	0.01	KRAS SIGNALING DN	-0.45	-1.87	0.000	0.00
PEROXISOME	0.30	1.41	0.011	0.04	ANGIOGENESIS	-0.55	-1.76	0.003	0.00
					HYPOXIA	-0.39	-1.67	0.000	0.00
					HEDGEHOG SIGNALING	-0.49	-1.61	0.007	0.00
					MYOGENESIS	-0.36	-1.53	0.002	0.01
					P53 PATHWAY	-0.35	-1.50	0.000	0.02
					APICAL SURFACE	-0.45	-1.50	0.018	0.02
**MATRIGEL VS SINGLE CELLS**									
**UPREGULATED**					**DOWNREGULATED**				
**HALLMARK PATHWAY**	**ES**	**NES**	**NOM p-val**	**FDR q-val**	**HALLMARK PATHWAY**	**ES**	**NES**	**NOM p-val**	**FDR q-val**
MYC TARGETS V1	0.64	3.43	0.000	0.00	INTERFERON GAMMA RESPONSE	-0.75	-3.12	0.000	0.00
E2F TARGETS	0.57	3.04	0.000	0.00	TNFA SIGNALING VIA NFKB	-0.70	-2.96	0.000	0.00
G2M CHECKPOINT	0.54	2.85	0.000	0.00	INTERFERON ALPHA RESPONSE	-0.77	-2.93	0.000	0.00
MYC TARGETS V2	0.64	2.78	0.000	0.00	ALLOGRAFT REJECTION	-0.63	-2.61	0.000	0.00
OXIDATIVE PHOSPHORYLATION	0.51	2.73	0.000	0.00	INFLAMMATORY RESPONSE	-0.60	-2.51	0.000	0.00
DNA REPAIR	0.46	2.32	0.000	0.00	IL6 JAK STAT3 SIGNALING	-0.63	-2.33	0.000	0.00
PROTEIN SECRETION	0.47	2.20	0.000	0.00	KRAS SIGNALING UP	-0.49	-2.01	0.000	0.00
UNFOLDED PROTEIN RESPONSE	0.43	2.16	0.000	0.00	COMPLEMENT	-0.49	-2.01	0.000	0.00
MTORC1 SIGNALING	0.39	2.06	0.000	0.00	IL2 STAT5 SIGNALING	-0.48	-1.98	0.000	0.00
GLYCOLYSIS	0.33	1.77	0.000	0.00	KRAS SIGNALING DN	-0.47	-1.94	0.000	0.00
REACTIVE OXYGEN SPECIES PATHWAY	0.40	1.68	0.009	0.00	APOPTOSIS	-0.48	-1.93	0.000	0.00
MITOTIC SPINDLE	0.28	1.46	0.000	0.02	COAGULATION	-0.47	-1.82	0.000	0.00
					HEDGEHOG SIGNALING	-0.57	-1.81	0.001	0.00
					MYOGENESIS	-0.41	-1.71	0.000	0.00
					HYPOXIA	-0.41	-1.68	0.000	0.00
					ANGIOGENESIS	-0.53	-1.67	0.006	0.00
					APICAL SURFACE	-0.49	-1.64	0.010	0.01
					EPITHELIAL MESENCHYMAL TRANSITION	-0.39	-1.61	0.000	0.02
					P53 PATHWAY	-0.35	-1.48	0.007	0.02
					NOTCH SIGNALING	-0.46	-1.46	0.052	0.00
**MYOGEL VS SINGLE CELLS**									
**UPREGULATED**					**DOWNREGULATED**				
**HALLMARK PATHWAY**	**ES**	**NES**	**NOM p-val**	**FDR q-val**	**HALLMARK PATHWAY**	**ES**	**NES**	**NOM p-val**	**FDR q-val**
MYC TARGETS V1	0.66	3.51	0.000	0.00	INTERFERON GAMMA RESPONSE	-0.73	-3.18	0.000	0.00
E2F TARGETS	0.62	3.30	0.000	0.00	INTERFERON ALPHA RESPONSE	-0.74	-2.97	0.000	0.00
G2M CHECKPOINT	0.60	3.20	0.000	0.00	TNFA SIGNALING VIA NFKB	-0.68	-2.95	0.000	0.00
MYC TARGETS V2	0.70	3.02	0.000	0.00	ALLOGRAFT REJECTION	-0.65	-2.81	0.000	0.00
OXIDATIVE PHOSPHORYLATION	0.56	3.01	0.000	0.00	INFLAMMATORY RESPONSE	-0.60	-2.60	0.000	0.00
UNFOLDED PROTEIN RESPONSE	0.49	2.36	0.000	0.00	IL6 JAK STAT3 SIGNALING	-0.59	-2.26	0.000	0.00
PROTEIN SECRETION	0.50	2.33	0.000	0.00	KRAS SIGNALING UP	-0.49	-2.15	0.000	0.00
DNA REPAIR	0.46	2.33	0.000	0.00	COMPLEMENT	-0.48	-2.06	0.000	0.00
MTORC1 SIGNALING	0.42	2.30	0.000	0.00	KRAS SIGNALING DN	-0.48	-2.03	0.000	0.00
MITOTIC SPINDLE	0.39	2.01	0.000	0.00	IL2 STAT5 SIGNALING	-0.46	-1.99	0.000	0.00
GLYCOLYSIS	0.34	1.77	0.000	0.00	APOPTOSIS	-0.44	-1.85	0.000	0.00
REACTIVE OXYGEN SPECIES PATHWAY	0.40	1.65	0.007	0.02	HEDGEHOG SIGNALING	-0.52	-1.73	0.009	0.00
FATTY ACID METABOLISM	0.29	1.47	0.012	0.03	MYOGENESIS	-0.40	-1.70	0.000	0.01
ADIPOGENESIS	0.26	1.42	0.007	0.00	COAGULATION	-0.41	-1.67	0.001	0.01
					HYPOXIA	-0.37	-1.58	0.000	0.02
					ANGIOGENESIS	-0.48	-1.58	0.016	0.02
					P53 PATHWAY	-0.35	-1.50	0.002	0.02
					APICAL SURFACE	-0.45	-1.48	0.023	0.00
					EPITHELIAL MESENCHYMAL TRANSITION	-0.34	-1.45	0.002	0.00

**Table 4:** **Significantly expressed hallmark pathways in freshly digested single cells compared to fresh tissue samples.** Results of the gene set enrichment analysis (GSEA) show the significantly upregulated hallmark pathways in freshly digested single cells compared to fresh tissue sample. The pathways that passed the filter criteria had FDR q-val < 0.05. ES, enrichment score; NES, normalized enrichment score

FRESHLY DIGESTED SINGLE CELLS VS. FRESH TISSUE				
UPREGULATED				
HALLMARK PATHWAY	ES	NES	NOM p-val	FDR q-val
TNFA SIGNALING VIA NFKB	0.70	3.57	0.000	0.00
ALLOGRAFT REJECTION	0.63	3.18	0.000	0.00
INFLAMMATORY RESPONSE	0.56	2.88	0.000	0.00
INTERFERON GAMMA RESPONSE	0.55	2.81	0.000	0.00
COMPLEMENT	0.54	2.76	0.000	0.00
IL6 JAK STAT3 SIGNALING	0.61	2.70	0.000	0.00
APOPTOSIS	0.54	2.66	0.000	0.00
IL2 STAT5 SIGNALING	0.50	2.58	0.000	0.00
HYPOXIA	0.50	2.50	0.000	0.00
KRAS SIGNALING UP	0.49	2.44	0.000	0.00
EPITHELIAL MESENCHYMAL TRANSITION	0.45	2.30	0.000	0.00
INTERFERON ALPHA RESPONSE	0.50	2.29	0.000	0.00
OXIDATIVE PHOSPHORYLATION	0.44	2.23	0.000	0.00
COAGULATION	0.46	2.15	0.000	0.00
UV RESPONSE UP	0.43	2.12	0.000	0.00
ADIPOGENESIS	0.40	2.06	0.000	0.00
MYC TARGETS V1	0.40	2.03	0.000	0.00
ANGIOGENESIS	0.54	1.94	0.000	0.00
REACTIVE OXYGEN SPECIES PATHWAY	0.48	1.87	0.000	0.00
ANDROGEN RESPONSE	0.40	1.83	0.002	0.00
UNFOLDED PROTEIN RESPONSE	0.37	1.74	0.000	0.00
P53 PATHWAY	0.33	1.68	0.000	0.01
XENOBIOTIC METABOLISM	0.33	1.66	0.000	0.01
HEME METABOLISM	0.32	1.58	0.000	0.03
PI3K AKT MTOR SIGNALING	0.31	1.46	0.006	0.03
UV RESPONSE DN	0.30	1.45	0.010	0.03
PEROXISOME	0.32	1.43	0.019	0.03
DNA REPAIR	0.29	1.43	0.014	0.03
TGF BETA SIGNALING	0.35	1.41	0.051	0.00

We have previously shown that culturing HNSCC cell lines on Myogel or Matrigel produces significant changes in many ontology groups compared to plastic. The same applies to the comparison between Myogel and Matrigel [14]. When we applied the same type of experiment here for patient-derived primary cells, the cells cultured on Matrigel had three significantly upregulated pathways and those were KRAS signaling UP, inflammatory response and allograft rejection, and 17 downregulated pathways including interferon alpha response, MYC targets V1, and epithelial mesenchymal transition and interferon gamma response, compared to plastic (Table 5; Fig. 2G). On the other hand, cells cultured on Myogel had two significant upregulated pathways and those were KRAS signaling UP and coagulation, and 5 downregulated pathways including interferon alpha response, interferon gamma response and epithelial mesenchymal transition, compared with plastic (Table 5; Fig. 2H). Cells cultured on Myogel had significantly more upregulated pathways (17 pathways) than downregulated (2 pathways) compared to cells cultured on Matrigel. Between the most important upregulated pathways we found MYC targets V2, MYC targets V1, oxidative phosphorylation, mTOR signaling, and epithelial mesenchymal transition (Table 5; Fig. 2I).

**Table 5:** **Significantly expressed Hallmark pathways in Matrigel and Myogel compared to plastic and Myogel compared to Matrigel.** Results of the gene set enrichment analysis. (GSEA) show the significantly expressed Hallmark pathways in different culturing conditions compared to fresh tissue sample. The pathways that passed the filter criteria had FDR q-val < 0.05. ES, enrichment score; NES, normalized enrichment score

MATRIGEL VS PLASTIC									
UPREGULATED					DOWNREGULATED				
HALLMARK PATHWAY	ES	NES	NOM p-val	FDR q-val	HALLMARK PATHWAY	ES	NES	NOM p-val	FDR q-val
KRAS SIGNALING UP	0.44	1.95	0.000	0.00	G2M CHECKPOINT	-0.55	-2.69	0.000	0.00
INFLAMMATORY RESPONSE	0.41	1.84	0.000	0.00	INTERFERON ALPHA RESPONSE	-0.59	-2.59	0.000	0.00
ALLOGRAFT REJECTION	0.37	1.69	0.000	0.01	E2F TARGETS	-0.52	-2.58	0.000	0.00
					UNFOLDED PROTEIN RESPONSE	-0.53	-2.41	0.000	0.00
					MITOTIC SPINDLE	-0.49	-2.38	0.000	0.00
					MYC TARGETS V1	-0.49	-2.37	0.000	0.00
					EPITHELIAL MESENCHYMAL TRANSITION	-0.44	-2.17	0.000	0.00
					MYC TARGETS V2	-0.53	-2.10	0.000	0.00
					MTORC1 SIGNALING	-0.40	-1.96	0.000	0.00
					UV RESPONSE DN	-0.42	-1.95	0.000	0.00
					APICAL JUNCTION	-0.38	-1.85	0.000	0.00
					INTERFERON GAMMA RESPONSE	-0.37	-1.77	0.000	0.01
					MYOGENESIS	-0.34	-1.62	0.000	0.02
					OXIDATIVE PHOSPHORYLATION	-0.31	-1.48	0.000	0.02
					ADIPOGENESIS	-0.31	-1.48	0.000	0.02
					ANDROGEN RESPONSE	-0.33	-1.47	0.011	0.02
					PROTEIN SECRETION	-0.33	-1.45	0.014	0.00
**MYOGEL VS PLASTIC**									
**UPREGULATED**					**DOWNREGULATED**				
**HALLMARK PATHWAY**	**ES**	**NES**	**NOM p-val**	**FDR q-val**	**HALLMARK PATHWAY**	**ES**	**NES**	**NOM p-val**	**FDR q-val**
KRAS SIGNALING UP	0.41	1.83	0.000	0.01	INTERFERON ALPHA RESPONSE	-0.61	-2.49	0.000	0.00
COAGULATION	0.44	1.79	0.000	0.01	INTERFERON GAMMA RESPONSE	-0.47	-2.15	0.000	0.00
					EPITHELIAL MESENCHYMAL TRANSITION	-0.39	-1.80	0.000	0.00
					MYOGENESIS	-0.36	-1.59	0.000	0.02
					ESTROGEN RESPONSE EARLY	-0.32	-1.47	0.002	0.04
**MYOGEL VS MATRIGEL**									
**UPREGULATED**					**DOWNREGULATED**				
**HALLMARK PATHWAY**	**ES**	**NES**	**NOM p-val**	**FDR q-val**	**HALLMARK PATHWAY**	**ES**	**NES**	**NOM p-val**	**FDR q-val**
G2M CHECKPOINT	0.54	2.68	0.000	0.00	INFLAMMATORY RESPONSE	-0.36	-1.72	0.000	0.01
E2F TARGETS	0.52	2.58	0.000	0.00	ALLOGRAFT REJECTION	-0.35	-1.65	0.000	0.01
MYC TARGETS V1	0.50	2.48	0.000	0.00					
MITOTIC SPINDLE	0.46	2.30	0.000	0.00					
OXIDATIVE PHOSPHORYLATION	0.44	2.18	0.000	0.00					
MYC TARGETS V2	0.52	2.09	0.000	0.00					
UNFOLDED PROTEIN RESPONSE	0.44	2.03	0.000	0.00					
UV RESPONSE DN	0.41	1.97	0.000	0.00					
MTORC1 SIGNALING	0.38	1.89	0.000	0.00					
PROTEIN SECRETION	0.40	1.77	0.000	0.00					
TGF BETA SIGNALING	0.42	1.65	0.007	0.01					
ANDROGEN RESPONSE	0.36	1.60	0.005	0.01					
ADIPOGENESIS	0.32	1.59	0.000	0.01					
EPITHELIAL MESENCHYMAL TRANSITION	0.30	1.53	0.002	0.02					
APICAL JUNCTION	0.31	1.52	0.002	0.02					
DNA REPAIR	0.31	1.47	0.009	0.03					
FATTY ACID METABOLISM	0.30	1.45	0.011	0.03					
SPERMATOGENESIS	0.32	1.45	0.010	0.03					

### Immune and ECM related genes are mostly affected by culturing cells on plastic, myogel, and matrigel

**Fig. 3:**
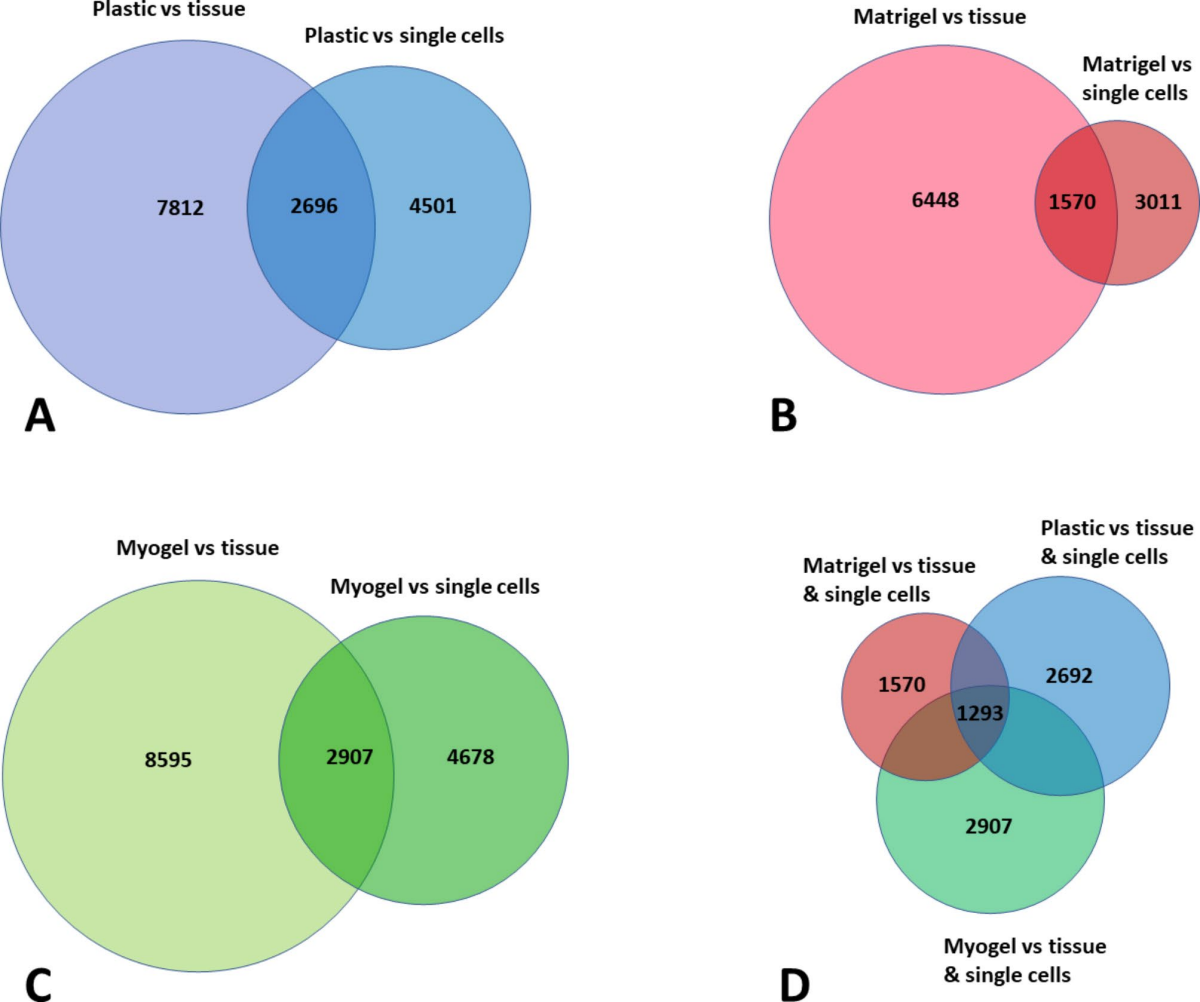
**Significantly differently expressed genes in cultured cells compared to fresh tissue sample and freshly isolated single cells.** Diagram shows the number of altered gene expressions in cultured cells in different culturing conditions (**A**) plastic, (**B**) Matrigel, and (**C**) Myogel, compared to fresh tissue and single cells. (**D**) Diagram shows significantly differently expressed genes in cells cultured on plastic, Matrigel, and Myogel compared to both fresh tissue samples and single cells. The genes that passed the filter criteria had a p < 0.05

RNA sequencing transcriptome profiles revealed significant changes in thousands of genes between cultured cells (on plastic, Myogel, and Matrigel) compared with fresh tissue and freshly digested single cells **(**Fig. 3A-C). The number of significantly differentially expressed genes (DE genes) between cultured cells and fresh tissue was almost double the number between cultured cells and freshly digested single cells (Fig. 3). Finally, as cultured cells clustered together in PCA, we compared the DE genes between different culturing conditions. We identified 1293 DE genes that were common between fresh tissue and freshly digested single cells together with the cells cultured either on plastic, Myogel, or Matrigel (Fig. 3D).

**Fig. 4:**
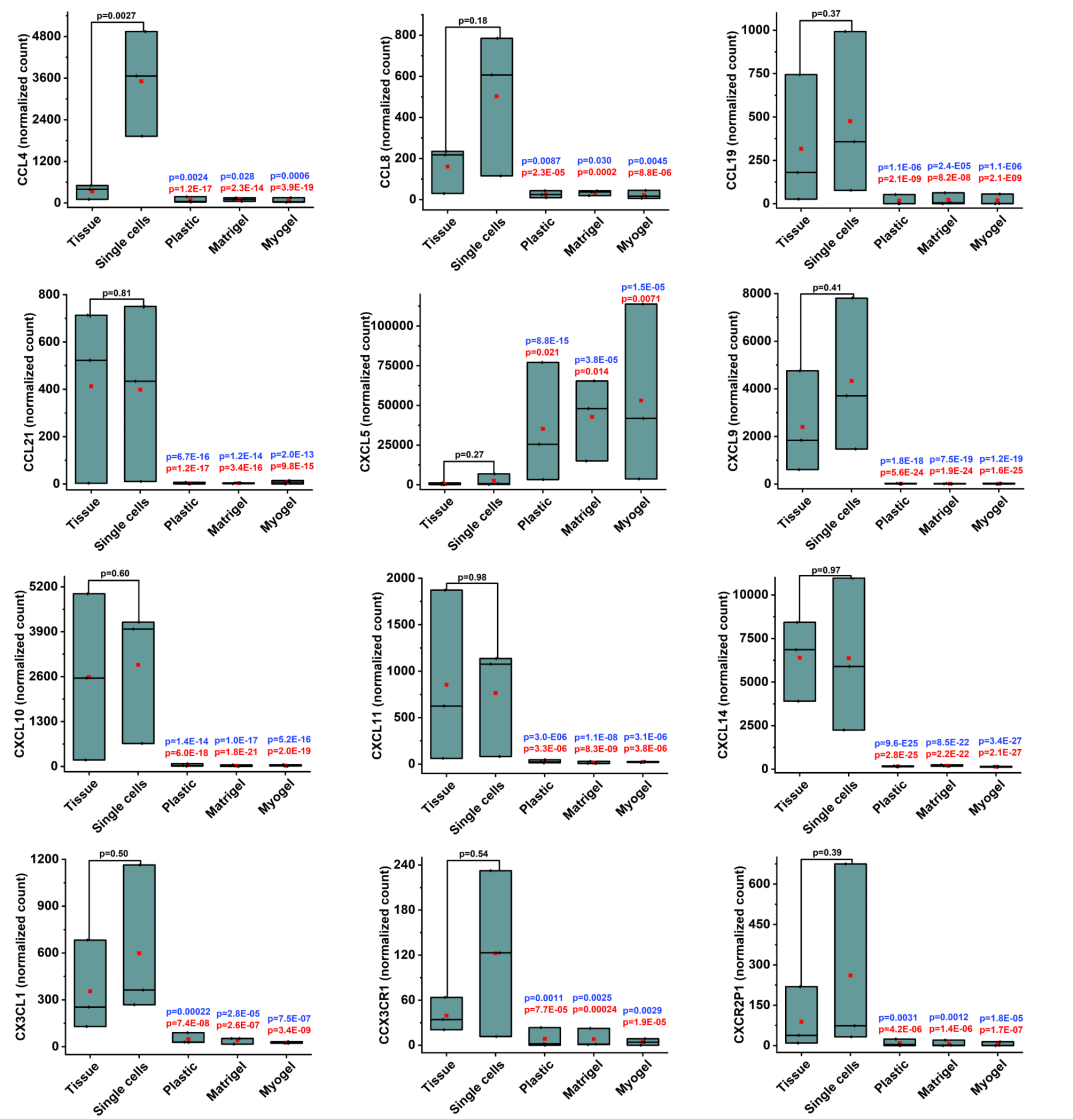
**Culturing patient-derived cells affect the expression of chemokine genes**. Differential expression (DE) analysis from RNA sequencing transcriptome profile revealed significant changes in the expression of genes related to chemokines in all culturing conditions compared to both fresh tissue sample and freshly isolated single cells. All molecules in this family, except CXCL5, were downregulated. The genes that passed the filter criteria had a p < 0.05. P values in blue represent the comparison with tissue, while the p values in red represent the comparisons with freshly isolated single cells. The mean is presented as a red box and the median as a black line

Out of these 1293 genes, we were able to identify 35 interesting genes that belong to five different gene families: chemokines and their receptors, matrix metalloproteinases (MMPs), collagen, the tumor necrosis factor (TNF) superfamily, and immune related molecules. The chemokines and their receptors family consisted of 12 molecules. All of them, except CXCL5, were downregulated in the cultured cells compared with fresh tissue and digested single cells (Fig. 4).

**Fig. 5:**
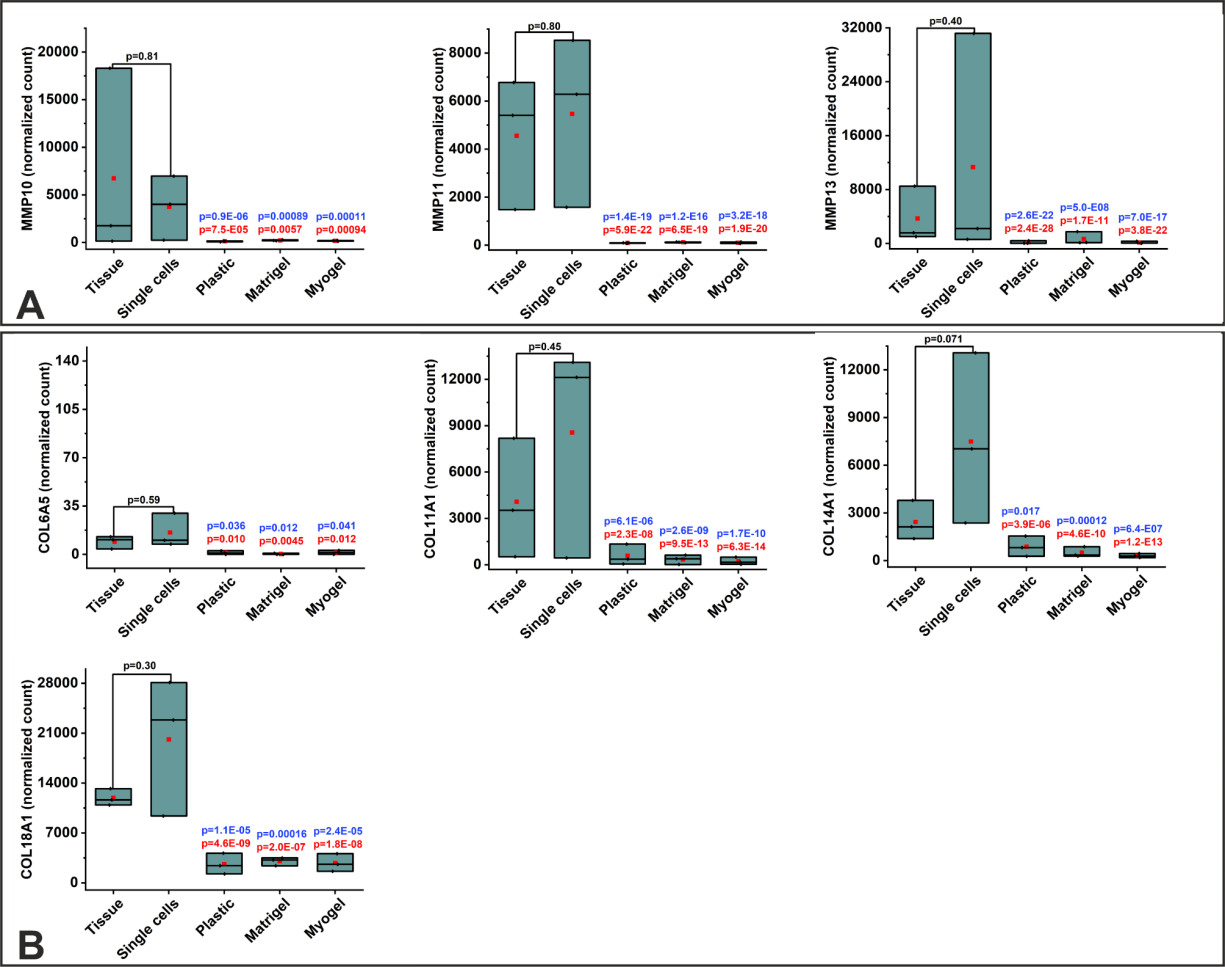
**Culturing patient-derived cells affect the expression of some MMP and collagen genes**. Differential expression (DE) analysis from RNA sequencing transcriptome profile revealed significant changes in the expression of genes in all culturing conditions compared to fresh tissue sample and freshly isolated cancer cells. (**A**) Three MMP genes were downregulated in cultured cells. (**B**) Four collagen genes were downregulated in cultured cells. The genes that passed the filter criteria had a p < 0.05. The mean is presented as a red box and the median as a black line

**Fig. 6:**
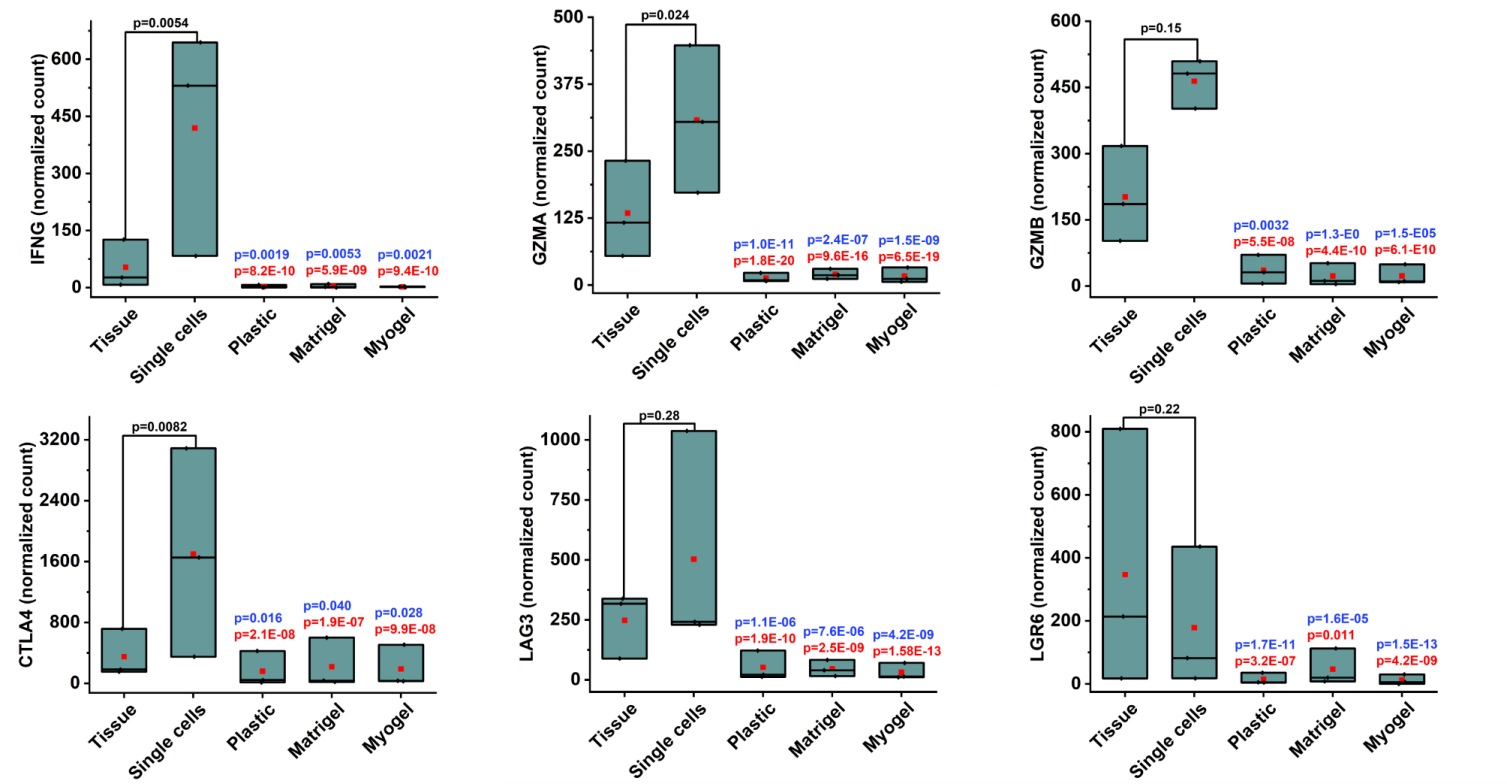
**Culturing patient-derived cells affects the expression of some genes related to the immune response**. Differential expression (DE) analysis from RNA sequencing transcriptome profile revealed significantly downregulated immune related genes in all culturing conditions compared to both fresh tissue sample and freshly isolated single cells. The genes that passed the filter criteria had a p < 0.05. The mean is presented as a red box and the median as a black line

**Fig. 7:**
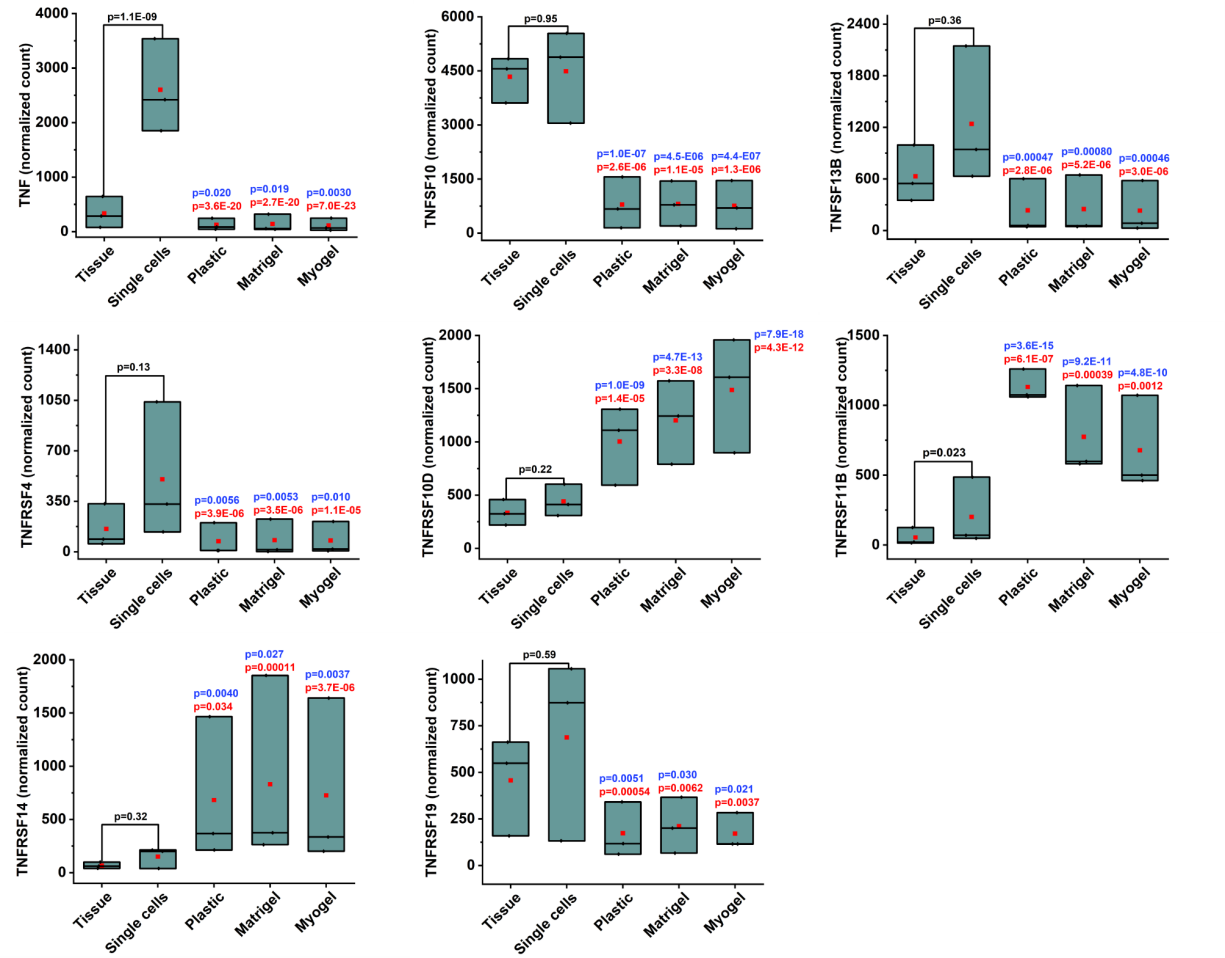
**Culturing patient-derived cells affect the expression of genes in TNF superfamily and TNF receptor superfamily**. Differential expression (DE) analysis from RNA sequencing transcriptome profile revealed significant changes in the expression of genes in TNE superfamily and TNF receptor superfamily in all culturing conditions compared to both fresh tissue sample and freshly isolated single cells. All TNF superfamily genes were downregulated. In the TNF receptor superfamily two genes were downregulated and three upregulated. The genes that passed the filter criteria had a p < 0.05. The mean is presented as a red box and the median as a black line

Similarly, families of MMP (MMP10, MMP11, MMP13), collagen (COL6A5, COL11A1, COL14A1, COL18A1), and immune related molecules (IFNG, GZMA, GZMB, CTLA4, LAG3, LGR6), were downregulated in the cultured cells compared with fresh tissue and digested single cells (Figs. 5 and 6). Additionally, we identified three differentially expressed genes in the TNF superfamily (TNF, TNFSF10, TNFSF13B) and five in the TNF receptor superfamily (TNFRSF4, TNFRSF10D, TNFRSF11B, TNFRSF14, TNFRSF19) (Fig. 7). All the three genes in the TNF superfamily were downregulated in the cultured cells compared with fresh tissue and digested single cells. On the other hand, three out of five genes from the TNF receptor superfamily were upregulated in the cultured cells compared with the fresh tissue and digested single cells (Fig. 7). Surprisingly, we detected only a few significantly differentially expressed genes when comparing cells cultured on plastic, Matrigel, and Myogel (Table 6).

**Table 6:** **DE genes in the comparison of different culturing conditions.** RNA transcriptome profiles were analysed for DE genes in cells cultured on Matrigel compared to plastic and on Myogel compared to Matrigel. The genes that passed the filter criteria had an adjusted p < 0.05. There were no DE genes comparing the transcriptomes of cells cultured on plastic and Myogel

MATRIGEL VS PLASTIC		
External gene name	log2FoldChange	padj
GSTA1	-20.2495	3.93E-05
RASD1	2.730824	0.013363
MEGF10	2.944972	0.023495
**MYOGEL VS MATRIGEL**		
**External gene name**	**log2FoldChange**	**padj**
COX6C	38.05927	7.63E-11
OR1Q1	-33.1441	4.00E-08
PSMC1P12	33.20884	4.19E-08
GSTA1	22,06886	5.17E-08
STMN4	31.407	2.19E-07
ZSCAN4	-29.6661	3.32E-06
LINC01645	28.28514	1.41E-05
CDY1	25.64111	0.000241
ZNF32-AS1	25.64111	0.000241
LINC02671	-24.7123	0.00057
ITPKB-AS1	24.47725	0.00067
Y_RNA	23.43644	0.001932
CELA1	-20.1613	0.004615
NPIPA9	-20.3217	0.034303
UBE2FP2	-20.2297	0.039927

### Differential responses to anti-cancer treatments on different matrices

**Fig. 8:**
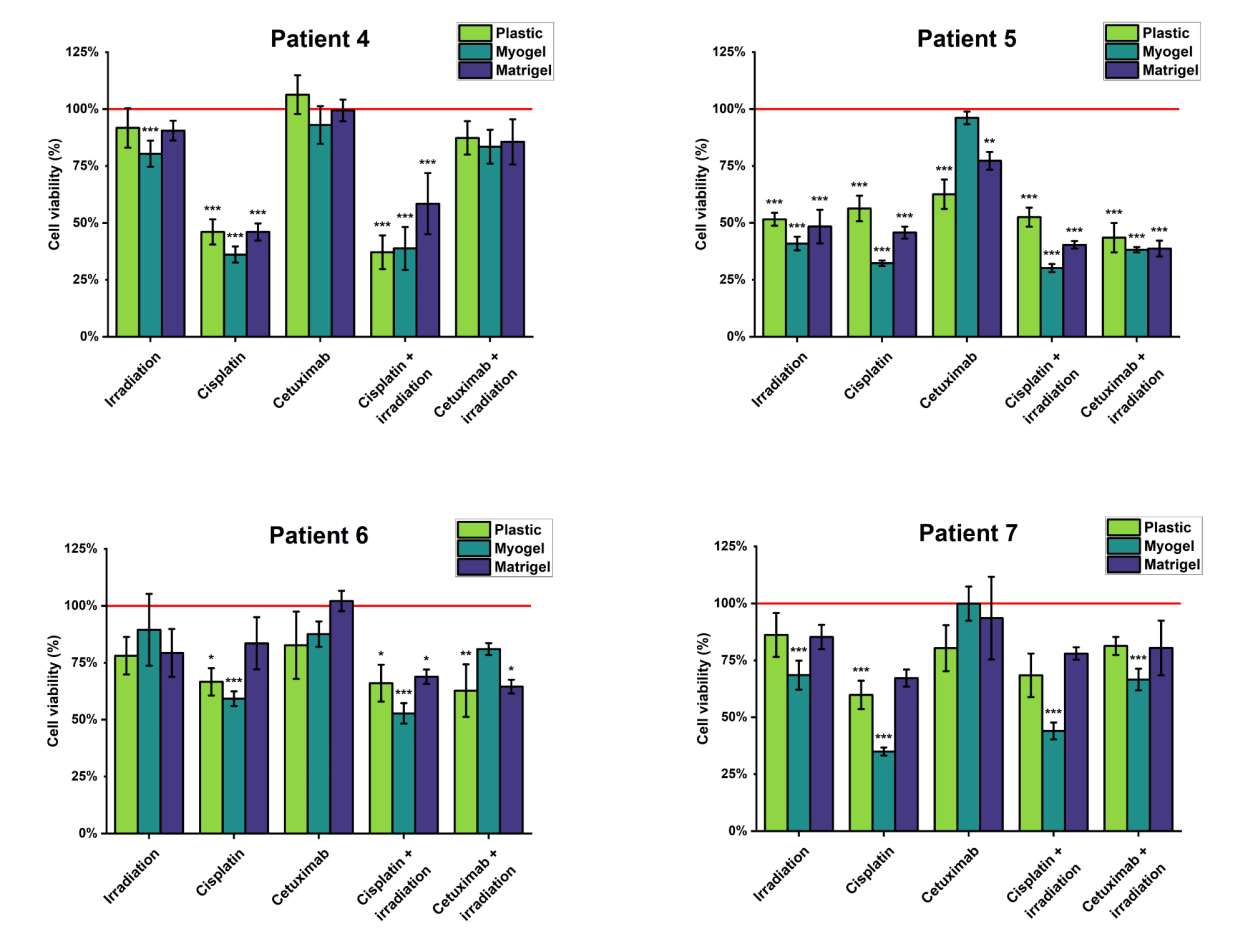
**Cell viability after anti-cancer treatment of cancer cells isolated from patient samples in different culturing conditions**. Cells isolated from patient samples were cultured in three different culturing conditions and treated with chemotherapy, and targeted therapy drug cetuximab with or without irradiation. The viability was measured using luminescent cell viability assay and normalized to the control (no treatment wells). The red line represents the cell viability values of the wells without any treatment. Data is presented as mean ± standard deviation of 9–18 wells. *P ≤ 0.05, ** ≤ 0.01

Freshly isolated cancer cells obtained from four HNSCC patients were cultured in three different culturing conditions and were subjected to anti-cancer treatments. Cell viability was measured to compare the cells’ treatment response in different culturing conditions (Fig. 8). For patient 4, all the five treatments setting gave a similar response on all the three culturing conditions, except for irradiation alone which was only effective when cells were cultured on Myogel (Fig. 8). Patient 5 results were similar to patient 4 with only one difference; the different responses between the culturing conditions were detected in cetuximab without irradiation (Fig. 8). We also detected differences in the range of activity of the anti-cancer treatments on different matrices. For example, cisplatin alone and irradiation plus cisplatin reduced cancer-cell viability by around 50% on plastic but this increased to 70% on Myogel (Fig. 8). In patient 6, differential responses were found in two treatment settings: cisplatin and irradiation plus cetuximab (Fig. 8). Patient 7 detected the largest differences in the response to the anti-cancer treatments, as the cells responded differently in four out of five treatment types showing the strongest responses primarily on Myogel (Fig. 8).

### The effect of freezing and re-culturing on cell response to anti-cancer treatments

**Fig. 9:**
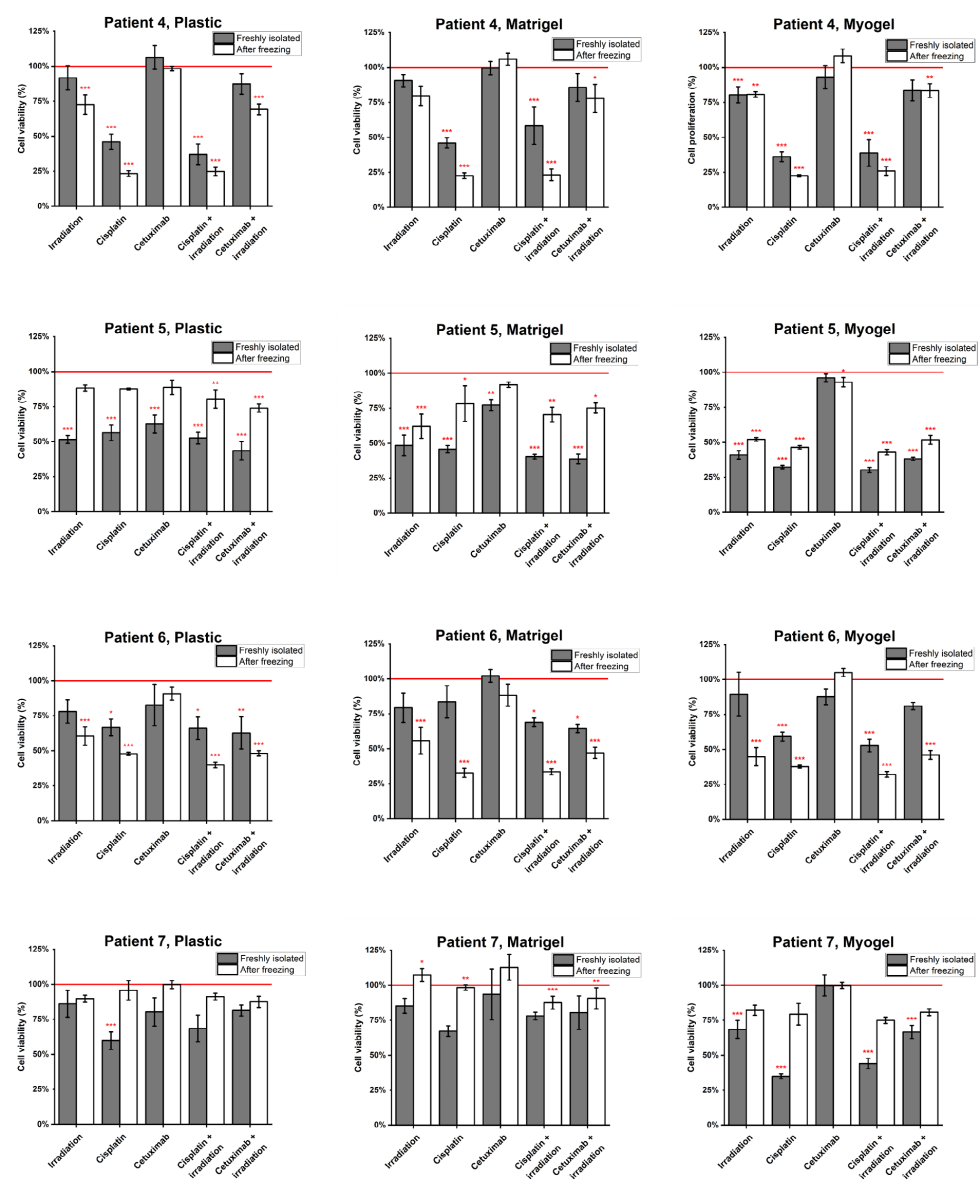
**Effect of anti-cancer treatments on cancer cells freshly isolated from patient samples and after one cycle of freezing and thawing**. Cancer cells were isolated from fresh patient samples and cultured in three different culturing conditions freshly and after freezing and thawing the cells. Cells were treated with chemotherapy drug cisplatin, targeted therapy drug cetuximab with or without irradiation, and the viability was measured using luminescent cell viability assay. The results were normalized to the control (no treatment) of each experiment. The red line represents the cell viability value of the wells without any treatment. Stars represent significant effect of the treatment compared to control value. Data is presented as mean ± standard deviation of 9–12 wells. *P ≤ 0.05, ** ≤ 0.01, *** ≤ 0.001

To investigate the effect of the freezing and re-culturing on cells response to anti-cancer treatments and if culturing matrices could manipulate these effects, we compared the cells response to the anti-cancer treatments between freshly obtained cells and the same cells after one cycle of freezing and thawing. For patient 4, the cells response to the anti-cancer treatments were similar before and after freezing in three out of five treatments in plastic and four out of five on Matrigel and Myogel **(**Fig. 9). Different responses were found in irradiation, for all three culturing conditions, and irradiation plus cetuximab for plastic (Fig. 9). A lower response to all the treatment settings after freezing and thawing was evident for patient 5, compared with freshly isolated cells on plastic and Matrigel, but not on Myogel **(**Fig. 9). In term of statistical significance, we observed a significantly different response for three treatment settings (irradiation, cisplatin, and cetuximab) between fresh and frozen cells when cultured on plastic and only one treatment setting (cetuximab) when cells were cultured on Matrigel or Myogel (Fig. 9). Patient 6’s cells response to the anti-cancer treatments was similar after thawing the cells in four out of five treatment setting in plastic and three out of five in Matrigel and Myogel. Likewise, for patient 7, cells cultured on plastic had similar response before and after freezing in four out of five treatments. However, in Matrigel and Myogel the treatment response was consistent in only one (cetuximab) out of five treatments.

## Discussion

HNSCC heterogeneity causes difficulty in treating these patients, and the selection of treatment options is generally guided by the primary tumor location, tumor stage, and pathologic characteristics [16]. Unfortunately, currently available treatments are largely ineffective; approximately 50% of patients experience recurrence and they are usually associated with severe toxicity [17], [18]. There is thus an unmet need for discovering new anti-cancer treatments as well as for methods to identify the patients that would best benefit from the current treatment of choice.

**Fig. 10:**
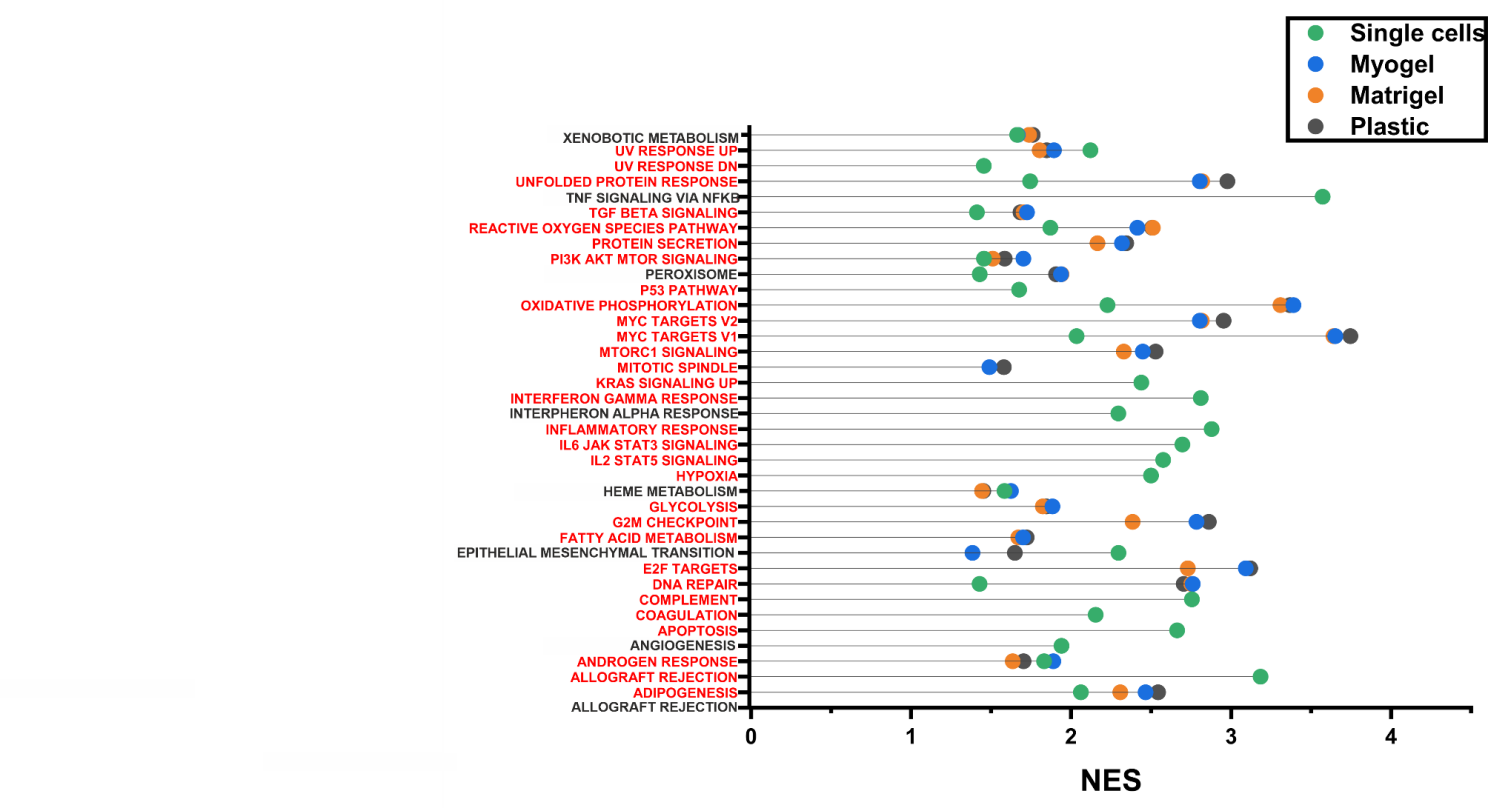
**Upregulated hallmark pathways shared with previous findings on pathways associated with collagenase dissociation**. Several of the upregulated hallmark pathways we found in cultured cells and freshly digested single cells compared to fresh tissue sample were also found to be associated with collagenase dissociation at 37 °C reported by O’Flanagan et al. [27]. The pathways that are marked in red are shared with the results from O’Flanagan et al. NES, normalized enrichment score

Many in vitro 3D assays have been developed using matrices extracted from human or animals, or derived from synthetic materials [19]. Previous studies have demonstrated that when adding the ECM component to cultures, cells behave differently compared to 2D in many features, including cell morphology, adhesion, polarity, differentiation, and gene expression [20] [21] [22] [23]. This study aimed to investigate how culturing conditions affect the transcriptomic profile of freshly isolated cancer cells and what conditions would best preserve the original cell gene expression. Furthermore, we studied how these changes affected the cancer cells’ response to anti-cancer treatments. Our hypothesis was that culturing freshly isolated cancer cells on ECM, Myogel, or Matrigel, will preserve the cells phenotype better compared to plastic. However, our results revealed large changes in the cultured cells transcriptomic profile in all the culturing conditions, plastic, Myogel, and Matrigel, with no superiority of one condition over the others. These results may be explained by the fact that in order to create single cells, the tumor sample must be subjected to dissociation and isolation [24]. The most common methods used for dissociation include enzymatic digestion with collagenase and mechanical dissociation [25]. The enzymatic and mechanical disruption of extracellular matrix and cell-cell contacts has been shown to impact the transcriptome of single cells [26]. Furthermore, solid tumors are heterogeneous in composition compared to isolated single cells. In addition to cancer cells, solid tumors are also composed of the TME, including endothelial cells, cancer-associated fibroblasts (CAFs), immune cells, and extracellular components [4]. Most of these cells are lost during the culturing process, and only cancer cells and CAFs usually remain. In a recent study by O’Flanagan et al. [27] the enzymatic dissociation with collagenase was observed to induce stress responses in patient-derived breast cancer xenografts. Furthermore, many genes and pathways were identified to be associated with the enzymatic digestion process and the stress reaction in cells [27]. Interestingly, many of the hallmark pathways that were associated with collagenase dissociationinduced stress responses found by O’Flanagan et al. were also detected in our results. We found 29 upregulated hallmark pathways when comparing freshly digested single cells with fresh tissue sample, and 21 of these pathways were also found in cells digested with collagenase by O’Flanagan et al. (Fig. 10) [27]. Furthermore, when comparing cultured cells (plastic, Matrigel, and Myogel) we found 22 pathways to be significantly upregulated and of these pathways 18 were found in the O’Flanagan et al. study (Fig. 10). Hence, the result of our study, together with previous findings, suggests that methods used for tumor dissociation are likely to change many gene expression profiles and thus influence the in vitro results, at least in the first three days of culturing from the time of digestion.

Gene ontology enrichment analysis (GSEA) showed that several hallmark pathways were upregulated in cultured cells compared with fresh tissue samples and most of them were shared between the different culturing conditions (plastic, Matrigel, and Myogel). We also found that in the comparison between cultured cells and freshly digested single cells, 12 pathways were shared in all culturing conditions and 11 of these were also found in the comparison between cultured cells and fresh tissue sample. Of these shared pathways, we identified several interesting ones, including MYC targets V1, MYC targets V2, and oxidative phosphorylation. MYC targets V1 and V2 include 200 and 58 genes, respectively, and they are associated with cell proliferation [28]. Upregulation of genes in these pathways has been associated with poor prognosis in different cancers as well as drug resistance, including HNSCC [29], [30], [31]. Oxidative phosphorylation involves 200 genes contributing to metabolic processes. Evidence suggests that high expression of oxidative phosphorylation genes is associated with better prognosis in OSCC as well as lung squamous cell carcinoma [28], [32].

We found androgen response and TGF beta signaling to be significantly upregulated in the comparison between cultured cells and fresh tissue samples, The androgen response pathway includes 117 genes associated with androgen receptor (AR) signaling [28]. AR acts as a master regulator of downstream androgen-dependent signaling pathway networks, and in addition to prostate and mammary glands, it is also expressed in oral mucosa [33]. Previous studies suggest that AR expression is associated with impaired prognosis in HNSCC [34], [35]. Conversely, TGF beta signaling has a dual role in cancer progression, as it works both as a tumor suppressor, but also contributes to processes promoting tumor progression [36], [37]. TGF beta signaling includes 54 genes that are known to play an important role in different cellular processes including cell proliferation, differentiation, apoptosis, and migration [28], [36]. Dysregulation of TGF beta signaling is common in many malignancies, including HNSCC [36], [37].

We found that PI3K/mTOR signaling was significantly upregulated in cultured cells compared to freshly digested single cells. The PI3K/Akt/mTOR pathway is a critical regulatory axis for cell growth, survival, motility, and metabolism in both normal physiology and cancer [38]. The members of the PI3K/Akt/mTOR axis interact with and contribute to the regulation of several other signaling molecules in HNSCC, including tumor suppressor protein p53, nuclear factor-kappa B (NF-κB), and mitogen-activated protein kinase (MAPK)/extracellular signal-regulated kinase (ERK) [38]. In HNSCC, the PI3K/AKT/mTOR pathway is upregulated in over 90% of both HPV positive and negative carcinomas, and upregulation of this pathway is associated with resistance to radio- and chemotherapy [39]. Interestingly, these pathways play a major role in drug discovery as they can be targeted by anti-cancer compounds. For example, temsirolimus is an mTOR inhibitor, which is approved for treating renal cell carcinoma and is in the clinical trials phase for HNSCC. Taking this into consideration, our results showing the extensive upregulation of these pathways in cultured cells compared to fresh tissue, could at least partially explain the failure of most anti-cancer compounds that pass in vitro testing.

We found 18 hallmark pathways to be significantly upregulated in cells cultured on Myogel, compared to Matrigel. Epithelial mesenchymal transition was one of the significantly upregulated pathways, which includes 200 genes [28]. Epithelial-to-mesenchymal transition (EMT) is a complex process whereby epithelial cells lose their characteristic features and promote a mesenchymal-like phenotype that determines stem cell behavior, metastasis formation, and wound healing [40], [41]. EMT is also a target for the treatment of HNSCC [40]. We have previously shown that Myogel is able to induce cancer cell invasion compared to other matrices used in cell cultures [14], [11]. This property is likely to be related to our results that showed the epithelial mesenchymal transition pathway to be upregulated in cells cultured on Myogel. Furthermore, EMT have been associated with treatment resistance in HNSCC, including resistance to EGFR inhibition and cisplatin [31]. In our previous study, we found that cells cultured on Myogel were more resistant to EGFR and MEK inhibitors than cells cultured on Matrigel [13]. Hence, these results indicate that cells cultured on Myogel provide more reliable results when testing anti-cancer drug effect.

RNA sequencing transcriptome profiling identified five different interesting gene families that were significantly different in all the culturing conditions compared to fresh tissue samples and freshly digested single cells. These were chemokines and their receptors, MMPs, collagen, TNF superfamily, and immune related molecules. Both collagen and MMPs are related to the ECM, where matrix metalloproteases (MMPs) are a collection of enzymes capable of cleaving ECM components, including collagen, and are related to various processes associated with tumor cell proliferation, angiogenesis, invasion, and metastasis [42]. HNSCCs are collagen-rich environments, and different collagen subtypes are expressed by both CAFs and malignant epithelial cells [43]. Moreover, collagen has been shown to promote proliferation and migration of HNSCC cells and it is associated with resistance to cisplatin [43]. Both MMPs and collagen genes were downregulated in cultured cells compared to fresh tissue samples and single cells. It is possible to hypothesize that the expression of these genes is affected by the digestion process the cells undergo. Out of the immune related molecules, including chemokines, the TNF superfamily, and IFN, most were downregulated in cultured cells compared to fresh tissue samples and freshly isolated single cells. These results are likely related to the enzymatic digestion and culturing of the cells, which leads to the loss of the innate and adaptive immune cells. This represents a new challenge in testing immunotherapy in an in vitro setting. Taken together, these results further confirm the fact that current methods used for in vitro cell cultures of patient-derived cells are not likely the most representative tumor models. However, we acknowledge the limitations of our study, including the fact that after 3–5 days of culturing isolated single cells we did not have pure cancer cells, and the culture also included small percentage of CAFs and immune cells. This must be taken in consideration when interpreting our gene expression results.

To investigate how cell culturing affects the cell phenotype in terms of their drug treatment response, we cultured freshly isolated cancer cells in different culturing conditions and treated them with anti-cancer treatments (cisplatin, cetuximab, and/or irradiation). In 12 out of 20 cases (60%), there was no difference in response to the anti-cancer treatment regardless of the matrix used; on the other hand, the rest, 40% showed a matrix dependent response to the anti-cancer therapies. These results, combined with our findings on the transcriptomic changes of freshly isolated and cultured cells in different conditions, indicates that reliable personalized drug response testing in vitro is challenging.

There is a trend of establishing cell lines when testing drug responses [44], and we wanted to investigate if the response is the same before and after freezing and re-culturing the cells. Cells cultured on plastic gave the same treatment response in 65% of cases (13/20), on Matrigel in 60% (12/20), and on Myogel in 55% of cases (11/20) before and after freezing, respectively. The composition of primary cultures varies, and fibroblast contamination is common [45]. CAFs adapt exceptionally well to the in vitro environment, and their rapid overgrowth is a challenge for preserving cancer cells [46]. The non-malignant cells in cell cultures might interfere with toxic drug response and cell viability assessment in our experiments. Hence, the well-established challenges with primary cell cultures likely influenced our results.

## Conclusion

Our study showed the limitations of in vitro drug testing using enzymatic digestion. To best of our knowledge, this is the first study to characterize the changes in gene expression after isolating and culturing HNSCC cells. Culturing patient-derived cells affect many pathways and thousands of genes and change the cells’ gene expression profile, which results in the cells not reflecting the actual patient response. A more complete and thorough understanding of how to preserve the transcriptome of the fresh tissue sample in cultured cells is needed to achieve more reliable results from in vitro studies with patient-derived cells. Furthermore, this knowledge is essential in developing reliable in vitro models avoiding the stress reaction for predicting patients’ treatment responses and advancing more personalized treatment approaches for HNSCC patients. Moreover, our results support our previous findings on Myogel, suggesting that culturing cells on human tumor-derived matrix promotes cell invasive properties.

## Data Availability

The data that support the findings of this study are available from the corresponding author, [TS], upon reasonable request.

## List of Abbreviations

HNSCC: Head and neck squamous cell carcinoma
TME: The tumor microenvironment
AR: Androgen receptor
CAFs: Cancer-associated fibroblasts
DE: Differential expression
ECM: Extracellular matrix
EMT: Epithelial-to-mesenchymal transition
ERK: Extracellular signal-regulated kinase
GSEA: Gene set enrichment analysis
MMPs: Matrix metalloproteinases
NF-κB: Nuclear factor-kappa B
TNF: Tumor necrosis factor
